# Synthesis and Electrochemical Proprieties of Novel Unsymmetrical Bis-Tetrathiafulvalenes and Electrical Conductivity of Their Charge Transfer Complexes with Tetracyanoquinodimethane (TCNQ)

**DOI:** 10.3390/ijms13077872

**Published:** 2012-06-25

**Authors:** Tahar Abbaz, Amel Bendjeddou, Abdelkrim Gouasmia, Zine Regainia, Didier Villemin

**Affiliations:** 1 Laboratory of Organic Materials and Heterochemistry, University of Tebessa, Constantine Road, Tebessa, 12000, Algeria; E-Mail: akgouasmia@hotmail.com; 2 Laboratory of Applied Organic Chemistry, Heterocyclic Chemistry Group, Department of Chemistry, Faculty of Science, University of Annaba, Annaba, 23000, Algeria; E-Mails: bendjeddouamel@gmail.com (A.B.); regainiadz@yahoo.fr (Z.R.); 3 Laboratory of Molecular and Thio-Organic Chemistry, UMR CNRS 6507, INC3M, FR 3038, Labex EMC3, ENSICAEN & University of Caen, 14050 Caen, France; E-Mail: didier.villemin@ensicaen.fr

**Keywords:** tetrathiafulvalenes, redox potentials, organic materials, conductivity

## Abstract

The synthesis and properties of a series of bis-tetrathiafulvalenes (bis-TTFs) containing nitrophenyl, aminophenyl or dimethylaminophenyl is reported. The synthesis was carried out by using routes involving Wittig-type, cross-coupling, reduction and alkylation reactions. The electron donor ability of these new compounds has been measured by cyclic voltammetry (CV). Charge transfer complexes with tetracyanoquinodimethane (TCNQ) were prepared by chemical redox reactions. The complexes have been proven to give conducting materials.

## 1. Introduction

Tetrathiafulvalene (TTF) and its derivatives have attracted attention for many years because of their electron donor ability and the electrical conductivity of their charge transfer salts, which was started in 1973 with the synthesis of the tetrathiafulvalene-tetracyanoquinodimethane complex (TTF-TCNQ) by Cowan and coworkers [1]. Since then, the progress made in the synthesis of such molecules has been closely related to the discovery of new materials [2] exhibiting conducting [3–6], superconducting [7,8], magnetic [9–11], or optical properties [12,13].

The TTF core and its derivatives—due to their characteristics, in particular their stability and reversible redox character—have found a significant number of applications in materials chemistry [14] such as molecular switches rotaxanes and catenanes [15], conductive materials [16] and superconductors [17], complex with the C_60_ [18], conductive polymers [19], materials for nonlinear optics [13], sponges cations [20], ferromagnetic organic magnets [9], liquid crystals [21], and dendrimers [22]. Our research is focused on the conducting and superconducting materials in the hope to improve results in the field and to aim to find an organic superconductor at room temperature.

Dimeric tetrathiafulvalenes are currently of interest to chemists for their applications in organic molecular materials. They can be divides into two types: one is linked through conjugated π-systems and the other through non-conjugated σ-chains [2].

In a continuation of previous work of our group [23–25], in this paper we now describe the synthesis and properties of some new unsymmetrically π-donors of bis-TTFs which linked directly by σ-bond with alkyl chains of different lengths, containing nitrophenyl, aminophenyl or dimethylaminophenyl groups, synthesized via cross-coupling, reduction and alkylation methods. The redox behavior of such precursors has been studied by cyclic voltammetry and finally the electrical conductivity of charge transfer complexes was measured.

## 2. Results and Discussion

**Scheme 1:**
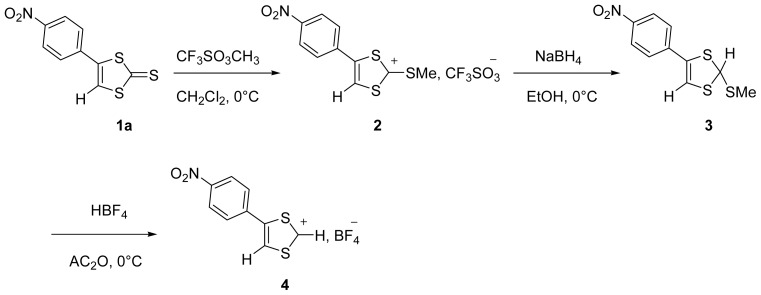
Synthetic route for the preparation of 4-(*p*-nitrophenyl)-1,3-dithiolium salt **4**.

Several steps are needed to convert the 4-(*p*-nitrophenyl)-1,3-dithiole-2-thione **1a** in the corresponding 4-(*p*-nitrophenyl)-1,3-dithiolium tetrafluoroborate **4** (Scheme 1).

The first step consists in methylating the thione **1a** [26] with methyl triflate in anhydrous CH_2_Cl_2_ at 0 °C. The desired salt, 2-methylthio-4-(*p*-nitrophenyl)-1,3-dithiolium trifluoromethane sulfonate **2**, isolated as a precipitate by simple addition of cold ether in 95% yield. During the second step, the salt **2** placed in suspension in ethanol is reduced by sodium borohydride at 0 °C. 2-methylthio-4-(*p*-nitrophenyl)-1,3-dithiole **3** is isolated as oil in 75% yield, which is treated directly in the next step. Finally, dethiomethylation of the reduced product **3** with tetrafluoroboric acid in acetic anhydride at 0 °C leads to 1,3-dithiolium **4** expected with a yield of 45%.

**Scheme 2:**
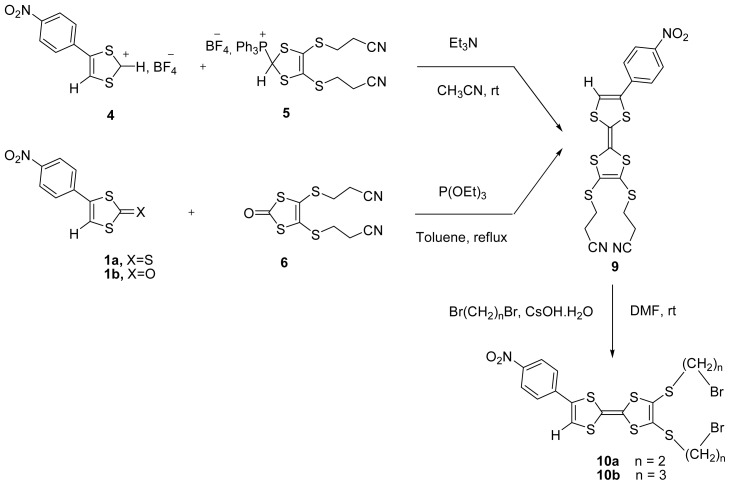
Synthetic route of mono-tetrathiafulvalenes (TTFs) **9** and **10**.

The synthesis of electron donors **9**, **10a** and **10b** based on a multi-step procedure were carried out as shown in Scheme 2.

We have synthesized 2,3-bis(2-cyanoethylthio)-6-*p*-nitrophenyl tetrathiafulvalene **9** by two different methodologies. The first involved a Wittig-type reaction using a weak base such as triethylamine which does not remove cyanoethyl protecting groups. The action of triethylamine in acetonitrile at room temperature on the phosphonium salt 4-(*p*-nitrophenyl)-1,3-dithiole-2-yl-triphenylphosphonium tetrafluoroborate **5** [27], generates the ylid, which reacts on the 1,3-dithiolium salt **4** to give the adduct intermediate. This is converted, by elimination of triphenylphosphine to 2,3-bis(2-cyanoethylthio)-6-*p*-nitrophenyl tetrathiafulvalene **9** in 30% yield.

The second route involved the reaction of chalcogenones 4-(*p*-nitrophenyl)-1,3-dithiole-2-thione **1a** or 4-(*p*-nitrophenyl)-1,3-dithiole-2-one **1b** [28] with 5-bis-(2-cyanoethylthio)-1,3-dithiole-2-one **6** [27], via a cross coupling method [29] in toluene at reflux in the presence of triethyl phosphite. As the reactivity of precursor 2-one was much higher than that of precursor 2-thione, mono-TTF **9** bearing two cyanoethylthio groups was obtained in 35% and 57% yield, respectively.

The deprotection of bis(2-cyanoethyl) groups of **9** with the aid of CsOH and sequentially reaction with 10 equivalents of 1,2-dibromoethane or 1,3-dibromopropane afforded the unsymmetrically functionalized mono-TTF derivatives 2,3-bis(2-bromoethylthio)-6-*p*-nitrophenyl tetrathiafulvalene **10a** or 2,3-bis(3-bromopropylthio)-6-*p*-nitrophenyl tetrathiafulvalene **10b** in 45% and 44% yield, respectively after purification by column chromatography.

**Scheme 3:**
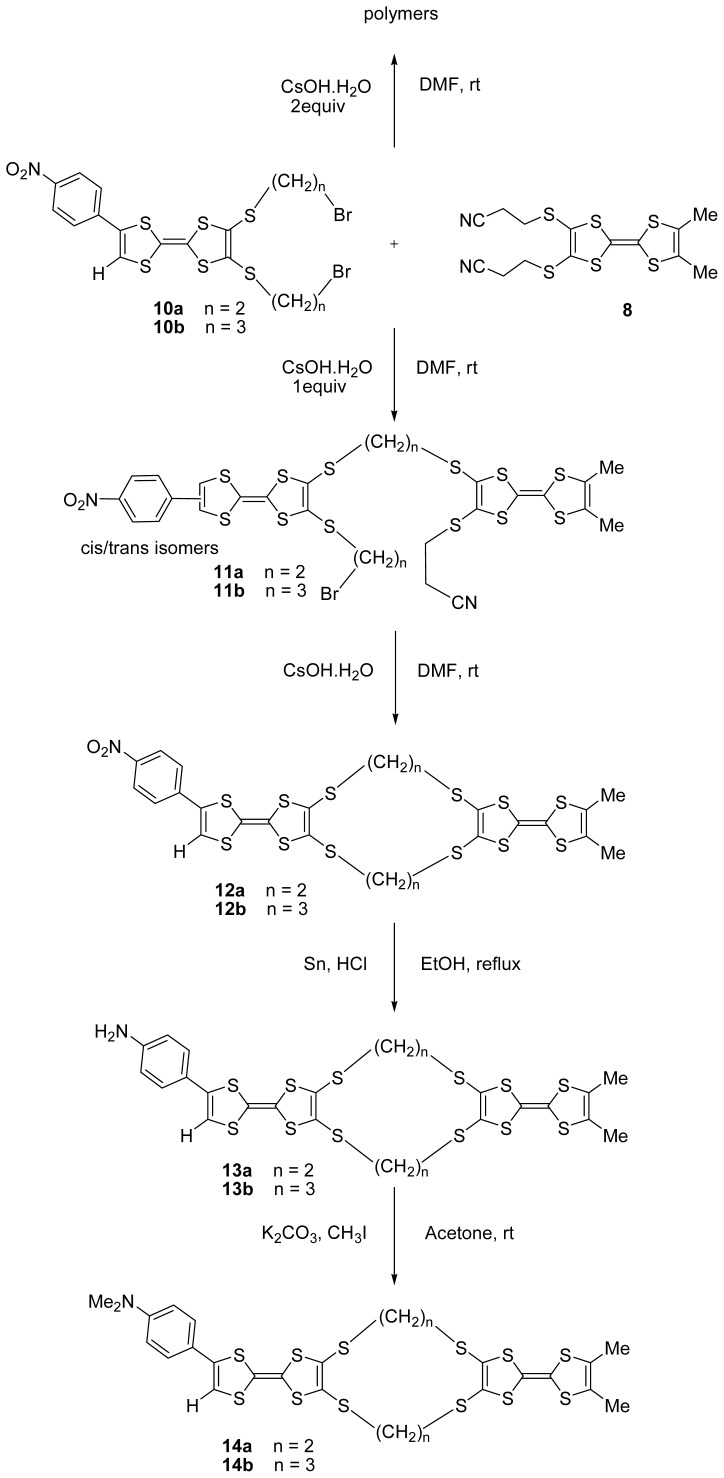
Synthetic route of bis-TTFs **11–14**.

The syntheses of bis-TTFs **11–14**, also based on a multi-step procedure, were carried out as shown in Scheme 3. The electrodonors **11a** and **11b** containing two TTF units were obtained in a mixture of *cis/trans* isomers in 40% and 38% yield, respectively, by using the reaction of **10a** or **10b** with 2,3-bis(2-cyanoethylthio)-6,7-di(methyl)tetrathiafulvalene **8** [30] using cesium hydroxide in DMF at room temperature. In this reaction, the risk of formation of a polymer is high, and to limit the polymerization it is necessary to work at high dilution (use a syringe pump). Thus, compounds **12a** and **12b** were obtained in 88% and 94% yield, respectively, by treatment of compounds **11a** or **11b** by cesium hydroxide in DMF at room temperature.

After, the nitro group of bis-TTFs **12a** or **12b** was reduced into an amino group in ethanol at reflux in the presence of tin and hydrochloric acid. The amino substituted bis-TTFs derivatives **13a** or **13b** were obtained after purification by column chromatography in 52% and 48% yield, respectively.

Finally the alkylation of amino bis-TTFs **13a** or **13b** was effected by treatment with K_2_CO_3_ (2 equiv.) and with 4 equivalents of iodomethane at room temperature, the dimethylamino bis-TTFs **14a** or **14b** were isolated by filtration and then extracted with CH_2_Cl_2_ and washed with water. **14a** and **14b** were obtained in 76% and 75% yields, respectively.

In the ^1^H NMR spectra of compound **9**, protons of CH=C exhibit a singlet at δ 6.82 ppm, in addition, protons of CH_2_CN showed a triplet at 2.78 ppm and 2.83 ppm, with coupling constants of 7.07 Hz and 6.00 Hz, respectively. Thus, the protons SCH_2_ showed a triplet at 3.14 ppm and 3.21 ppm, with coupling constants of 7.07 Hz and 6.00 Hz, respectively. The ^1^H NMR spectra of the **10a**,**b** revealed the absence of the CH_2_CH_2_CN group protons and the presence of (CH_2_)*_n_*Br protons. **10a** showed two triplets at 3.30 ppm and at 3.85 ppm with coupling constants of 6.72 Hz and 6.33 Hz, respectively. **10b** showed a multiplet at 2.15 ppm and two triplets at 2.93 ppm and at 3.55 ppm, with coupling constants of 6.78 Hz and 6.36 Hz, respectively. Further confirmation for the structure of **12a**,**b** was obtained from their mass spectral data, where they showed ion peaks at [M + H]^+^ 739 and [M + H]^+^ 767, respectively, and by their ^1^H RMN spectra, the aromatic protons for **12a** as two doublets at 7.55 ppm and at 8.25 ppm, with the same coupling constants of 9.37 Hz, as well as another characteristic triplet at 3.59 ppm for the two (CH_2_)_2_ groups protons. On the other hand, the spectrum of **12b** exhibited a multiplet at 2.47 ppm for the two CH_2_CH_2_CH_2_ groups. Moreover, the spectra of **13a**,**b** showed amino group protons as multiplet around 3.50–3.85 ppm. The final products **14a**,**b** showed the absence of the amino group proton signals and the presence of dimethylamino group proton signals as singlets at 3.35 ppm and at 3.20 ppm, respectively.

### 2.1. Electrochemical Studies

The redox behavior of these new functional mono- and bis-TTF was studied in solution by cyclic voltammetry (CV) and by square wave voltammetry (SQW). Measurements were performed under nitrogen at room temperature using a glassy carbon working electrode, a Pt counter electrode and a standard calomel electrode (SCE) as reference, with tetrabutylammonium perchlorate (*n*-Bu_4_NClO_4_, 0.1 M) in dry acetonitrile, as supporting electrolyte. A scan rate of 100 m·Vs^−1^ was used. The CV measurements showed reversible redox waves for all the compounds studied and the corresponding oxidation potentials Eox were determined by the SQW technique. The results are summarized in Table 1.

**Figure 1:**
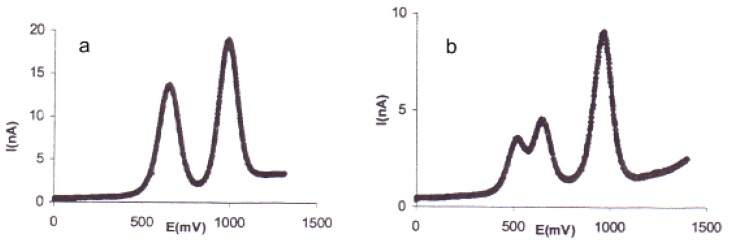
Voltammogram of mono-TTF **9** (type **I**) (**a**) and bis-TTF **14a** (type **II**) (**b**).

The type **I** (**a**) SQW curve shows two sharp oxidation waves each with one electron process for the mono-TTF (Figure 1a). This kind of voltammogramm is observed for compounds **9**, **10a** and **10b**. The type **II** (**b**) SQW curve is observed for compounds **11a**,**b**, **12a**,**b**, **13a**,**b** and **14a**,**b** (Figure 1b). We can clearly see three oxidation peaks with respectively a 1, 1 and 2 electron process. The real distinction of the two first oxidation waves is clearly due to the difference of the two TTF units of the bis-TTFs.

The oxidation potentials are almost identical for each pair of bis-donors (**11a**, **11b**), (**12a**, **12b**), (**13a**, **13b**) and (**14a**, **14b**). The first oxidation potentials in each pair are shifted cathodically by 20 mV. This may be due to the alkyl linked group. The oxidation potentials of compounds **14a**,**b** are slightly higher than that of compounds **13a**,**b**, on the other hand, the compounds **12a**,**b** are slightly higher than that of compounds **14a**,**b**. This should be attributable to the electronic properties of the nitrophenyl, aminophenyl and dimethylaminophenyl groups. All the oxidation potential values measured for mono-TTFs were found higher than the oxidation potentials of bis-TTFs. These results showed the good donor ability of this new series of mono-TTFs derivatives, which consequently should lead to conducting materials.

### 2.2. Preparation and Electrical Conductivity of Charge Transfer Complexes

The first report on the electrical conductivity in an organic solid appeared in 1954 [31], namely, a perylene—bromine complex, which has a room-temperature conductivity of 0.1 S cm^−1^. In 1960, the organic acceptor TCNQ [32] was synthesized as well as a great number of its conducting charge-transfer complexes and radical ion salts.

In the 1970s, the organic donor TTF [33] led to the first organic metal TTF-TCNQ [1]. Its room-temperature conductivity (500 S cm^−1^) increases with a decrease of the temperature to the value of 6000 S cm^−1^ at 60 K where a metal-insulator transition occurs. Since then, great interest has been devoted to this type of material, and a great number of new organic donors and acceptors have been synthesized as well as their charge-transfer salts.

Therefore, the complexation of the donors **9**–**14** with 7,7,8,8-tetracyanoquinodimethane (TCNQ) in hot acetonitrile solution gave the corresponding charge transfer complexes (CTC). Most of the solids were isolated in powder forms. Electrical conductivity was only measured on compressed pellets at room temperature using a two probe technique. The results are reported in Table 2.

For this family of materials, only CTC **9-TCNQ**, **10a-TCNQ** and **10b-TCNQ** resulting from mono-TTFs, can be classified in the area of conductors. In fact, they have a conductivity measured on powder compressed pellets of 8.60 10^−1^ to 1.30 10^−2^ S cm^−1^, that allows a conductivity ten times greater on single crystal.

Other, CTC resulting from bis-TTFs can be classified in the category of semi-conductors materials with conductivities from 10^−3^ to 10^−6^ S cm^−1^. This can be due to a structural disorder and/or a full charge transfer of an electron for each molecule.

## 3. Experimental Section

### 3.1. General

NMR spectra were recorded on a Brucker AC 250 instrument. Microanalyses were performed in the Microanalysis Laboratory of ENSCM (Montpellier). FAB mass spectra were recorded on a JOEL JMS-DX 300 spectrometer. Uncorrected melting points were measured on a 510 Buchi apparatus. Cyclic voltammetry measurements were carried out on a PAR-273 potentiostat/galvanostat. All solvents were dried by standard methods and all commercial reagents used without purification. All reactions were performed under an inert atmosphere of nitrogen.

#### 3.2. 2-Methylthio-4-(*p*-nitrophenyl)-1,3-dithiolium Trifluoromethane Sulfonate **2**

A suspension of 4-(*p*-nitrophenyl)-1,3-dithiole-2-thione **1a** (4.28 g, 16.81 mmol) in dry methylene chloride (25 mL) was treated with methyl triflate (2.4 mL, 19.2 mmol) at 0 °C. The mixture was stirred under nitrogen for 4 h and dry ether (150 mL) was added. The violet-orange salt **2** was obtained by filtration after 24 h, washed with more dry ether, and dried. Yield = 95%; orange powder, mp = 237 °C; ^1^H NMR (CDCl_3_) δ ppm: 3.10 (s, 3H, CH_3_S); 7.37 (s, 1H, HC-S); 7.53 (m, 2H_arom_); 8.24 (m, 2H_arom_); M.S: (NOBA, FAB > 0): 420 [M + H]^+^; M = 419; Anal. Calcd for C_11_H_8_S_4_NO_5_F_3_: C, 31.49; H, 1.92; S, 30.57; found: C, 31.70; H, 2.14; S, 30.60.

#### 3.3. 2-Methylthio-4-(*p*-nitrophenyl)-1,3-dithiole **3**

A suspension of **2** (7.73 g, 18.45 mmol) in dry ethanol (10 mL) was treated with NaBH_4_ (3.41 g, 92.25 mmol) at 0 °C under nitrogen. After the reaction mixture was stirred for 2 h, the solvent was evaporated under vacuum. The residue was dissolved in dichloromethane (100 mL), washed three times with water and dried over magnesium sulphate; the product was obtained after evaporation. Yield = 75%; TLC: Rf = 0.85 (CH_2_Cl_2_); dark red oil; ^1^H NMR (CDCl_3_) δ ppm: 2.84 (s, 3H, CH_3_S); 5.63 (s, 1H); 7.15 (s, 1H, HC-S); 7.33 (m, 2H_arom_); 8.00 (m, 2H_arom_); M.S: (NOBA, FAB > 0): 272 [M + H]^+^; M = 271; Anal. Calcd for C_10_H_9_S_3_NO_2_: C, 44.25; H, 3.34; S, 35.44; found: C, 44.00; H, 3.07; S, 35.75.

#### 3.4. 4-(*p*-Nitrophenyl)-1,3-dithiolium Tetrafluoroborate **4**

Tetrafluoroboric acid (0.92 g, 10.60 mmol) was added dropwise under nitrogen to a solution of **3** (2.61g, 9.64 mmol) in 30 mL of acetic anhydride at 0 °C. The reaction mixture was stirred for 15 min, then anhydrous ether was added. The 1,3-dithilium salt was precipitated, collected by filtration and washed with ether. Yield = 45%; white powder, mp = 253 °C; ^1^H NMR (CDCl_3_) δ ppm: 7.50 (m, 2H_arom_); 7.85 (s, 1H, HC=C-S); 8.20 (m, 2H_arom_); 9.35 (s, 1H); M.S: (NOBA, FAB > 0): 312 [M + H]^+^; M = 311; Anal. Calcd for C_9_H_6_S_2_NO_2_BF_4_: C, 34.74; H, 1.94; S, 20.61; found: C, 34.98; H, 2.16; S, 20.96.

#### 3.5. 2,3-Bis(2-cyanoethylthio)-6-*p*-nitrophenyl Tetrathiafulvalene **9**

##### 3.5.1. Method 1

4-(*p*-Nitrophenyl)-1,3-dithiole-2-thione **1a** [26] or 4-(*p*-nitrophenyl)-1,3-dithiole-2-one **1b** [28] and 5-bis-(2-cyanoethylthio)-1,3-dithiole-2-one **6** [27] were synthesized as described in the literature. Under a nitrogen atmosphere, 25 mL of freshly distilled triethyl phosphite was added to the mixture of **1a** (0.5 g, 1.96 mmol) or **1b** (0.5 g, 2.09 mmol) and **6** (1 equiv.). The resulting mixture was heated over an oil bath up to 110 °C and stirred for a further 4 h. The solvent was then removed under reduced pressure. Compound **9** was obtained by column chromatography of the residue (silica gel, eluting with dichloromethane and petroleum ether (2:1)) in 35% and 57% yield, respectively.

##### 3.5.2. Method 2

A solution of the dithiolium salt **4** (0.5 g, 1.60 mmol) and (4,5-bis((2-cyanoethyl)thio)-1,3-dithiol-2-yl)triphenylphosphonium tetrafluoroborate **5** (0.99 g, 1.60 mmol) in acetonitrile (30 mL) was treated with triethylamine (0.27 mL, 1.92 mmol) at room temperature under nitrogen and the reaction mixture was stirred for 4 h, the solvent was evaporated under vacuum. The residue was purified by column chromatography on silica gel with dichloromethane and petroleum ether (2:1) as the eluent to afford **9** in 30% yield.

TLC: Rf = 0.53 (CH_2_Cl_2_/petroleum ether) (2:1); dark-violet powder, mp = 175 °C; ^1^H NMR (CDCl_3_) δ ppm: 2.78 (t, 2H, CH_2_S, *J* = 7.07 Hz); 2.83 (t, 2H, CH_2_S, *J* = 6.00 Hz); 3.14 (t, 2H, CH_2_CN, *J* = 7.07 Hz); 3.21 (t, 2H, CH_2_CN, *J* = 6.00 Hz); 6.82 (s, 1H, C=CH); 7.40 (d, 2H_arom_, *J* = 9.00 Hz); 8.28 (d, 2H_arom_, *J* = 9.00 Hz); M.S: (NOBA, FAB > 0): 496 [M + H]^+^; M = 495; Anal. Calcd for C_18_H_13_S_6_N_3_O_2_: C, 43.61; H, 2.64; S, 38.81; found: C, 43.40; H, 2.40; S, 39.06.

### 3.6. Synthesis of Mono-TTFs **10a** and **10b**

Cesium hydroxide monohydrate (0.62 g, 3.72 mmol) in dry methanol (10 mL) was added to tetrathiafulvalene dicyano derivative **9** (0.5 g, 1.69 mmol) dissolved in dry and degassed DMF (30 mL). The reaction mixture was stirred for 10 min, the colour becoming dark violet. Then, an excess of 1,2-dibromoethane or 1,3-dibromopropane (10 equiv.) was added in one portion. The colour of the reaction mixture became clear, and the reaction mixture was stirred at room temperature for 1 h. The solvent was removed *in vacuo*, the residue was dissolved in dichloromethane (100 mL), washed three times with water and dried over magnesium sulphate. The mixture was concentrated *in vacuo* and the residue was purified by chromatography on a silica gel column (eluent: dichloromethane).

*2,3-Bis(2-bromoethylthio)-6-*p*-nitrophenyl tetrathiafulvalene* ***10a***: Yield = 45%; TLC: Rf = 0.85 (CH_2_Cl_2_); dark-violet powder, mp = 93 °C; ^1^H NMR (CDCl_3_) δ ppm: 3.30 (t, 4H, SCH_2_, *J* = 6.72 Hz); 3.85 (t, 4H, CH_2_Br, *J* = 6.33 Hz); 6.80 (s, 1H, C=CH); 7.55 (d, 2H_arom_, *J* = 9.28 Hz); 8.17 (d, 2H_arom_, *J* = 9.28 Hz); M.S: (NOBA, FAB > 0): 604 [M + H]^+^; M = 603; Anal. Calcd for C_15_H_14_S_6_NO_2_Br: C, 31.84; H, 2.17; S, 31.88; found: C, 32.09; H, 2.37; S, 31.73.

*2,3-Bis(3-bromopropylthio)-6-*p*-nitrophenyl tetrathiafulvalene* ***10b***: Yield = 44%; TLC: Rf = 0.85 (CH_2_Cl_2_); dark-violet powder, mp = 84 °C; ^1^H NMR (CDCl_3_) δ ppm: 2.15 (m, 4H, SCH_2_*CH_2_*CH_2_Br, *J* = 6.58 Hz); 2.93 (t, 4H, SCH_2_, *J* = 6.78 Hz); 3.55 (t, 4H, CH_2_Br, *J* = 6.36 Hz); 6.76 (s, 1H, C=CH); 7.50 (d, 2H_arom_, *J* = 9.38 Hz); 8.20 (d, 2H_arom_, *J* = 9.38 Hz); M.S: (NOBA, FAB > 0): 632 [M + H]^+^; M = 631; Anal. Calcd for C_16_H_16_S_6_NO_2_Br: C, 34.23; H, 2.71; S, 30.46; found: C, 34.03; H, 2.56; S, 30.76.

### 3.7. Synthesis of Bis-TTFs **11a** and **11b**

Compounds **11a** and **11b** were synthesized by employing the same experimental process as **10** from 1 equiv. of **10a** or **10b**, 1 equiv. of 2,3-bis(2-cyanoethylthio)-6,7-di(methyl)tetrathiafulvalene **8** and 1 equiv. of cesium hydroxide.

p*-Nitrophenyl bis-tetrathiafulvalene* ***11a***: Yield = 40%; TLC: Rf = 0.74 (CH_2_Cl_2_); violet powder, mp = 104 °C; ^1^H NMR (CDCl_3_) δ ppm: 1.84 (s, 6H, C=CMe); 2.84 (t, 2H, CH_2_S, *J* = 7.05 Hz); 3.19 (t, 2H, CH_2_CN, *J* = 7.05 Hz); 3.37 (t, 2H, SCH_2_, *J* = 6.70 Hz); 3.89 (t, 2H, CH_2_Br, *J* = 6.35 Hz); 3.62 (t, 4H, SCH_2_, *J* = 7.37 Hz); 6.85 (s, 1H, C=CH); 7.56 (d, 2H_arom_, *J* = 9.39 Hz); 8.26 (d, 2H_arom_, *J* = 9.39 Hz); M.S: (NOBA, FAB > 0): 869 [M + H]^+^; M = 868; Anal. Calcd for C_27_H_19_S_12_N_2_O_2_Br: C, 37.35; H, 2.21; S, 44.32; found: C, 37.60; H, 2.46; S, 44.12.

p*-Nitrophenyl bis-tetrathiafulvalene* ***11b***: Yield = 38%; TLC: Rf = 0.74 (CH_2_Cl_2_); violet powder, m.p. = 96 °C; ^1^H NMR (CDCl_3_) δ ppm: 1.80 (s, 6H, C=CMe); 2.81 (t, 2H, CH_2_S, *J* = 7.05 Hz); 3.16 (t, 2H, CH_2_CN, *J* = 7.05 Hz); 2.09 (m, 2H, SCH_2_*CH_2_*CH_2_Br, *J* = 6.56 Hz); 2.88 (t, 2H, SCH_2_, *J* = 6.74 Hz); 3.52 (t, 2H, CH_2_Br, *J* = 6.30 Hz); 2.45 (m, 2H, SCH_2_*CH_2_*CH_2_S); 2.95 (t, 4H, SCH_2_); 6.82 (s, 1H, C=CH); 7.54 (d, 2H_arom_, *J* = 9.39 Hz); 8.24 (d, 2H_arom_, *J* = 9.39 Hz); M.S: (NOBA, FAB > 0): 897 [M + H]^+^; M = 896; Anal. Calcd for C_29_H_23_S_12_N_2_O_2_Br: C, 38.87; H, 2.59; S, 42.93; found: C, 39.07; H, 2.80; S, 42.74.

### 3.8. Synthesis of Bis-TTFs **12a** and **12b**

Compounds **12a** and **12b** were synthesized by employing the same experimental process as **10** from 1 equiv. of **11a** or **11b** and 1 equiv. cesium hydroxide.

p*-Nitrophenyl bis-tetrathiafulvalene* ***12a***: Yield = 88%; TLC: Rf = 0.70 (CH_2_Cl_2_); violet powder, mp = 163 °C; ^1^H NMR (CDCl_3_) δ ppm: 1.55 (s, 6H, C=CMe); 3.59 (t, 8H, SCH_2_, *J* = 7.40 Hz); 6.83 (s, 1H, C=CH); 7.55 (d, 2H_arom_, *J* = 9.37 Hz); 8.25 (d, 2H_arom_, *J* = 9.37 Hz); M.S: (NOBA, FAB > 0): 739 [M + H]^+^; M = 738; Anal. Calcd for C_24_H_21_S_12_NO_2_: C, 39.05; H, 2.59; S, 52.12; found: C, 39.30; H, 2.69; S, 51.92.

p*-Nitrophenyl bis-tetrathiafulvalene* ***12b***: Yield = 94%; TLC: Rf = 0.70 (CH_2_Cl_2_); violet powder, m.p. = 155 °C; ^1^H NMR (CDCl_3_) δ ppm: 1.55 (s, 6H, C=CMe); 2.47 (m, 4H, SCH_2_CH_2_CH_2_S); 2.99 (t, 8H, SCH_2_); 6.83 (s, 1H, C=CH); 7.55 (d, 2H_arom_, *J* = 9.39 Hz); 8.25 (d, 2H_arom_, *J* = 9.39 Hz); M.S: (NOBA, FAB > 0): 767 [M + H]^+^; M = 766; Anal. Calcd for C_25_H_23_S_12_NO_2_: C, 40.75; H, 3.02; S, 50.21; found: C, 40.95; H, 3.17; S, 49.96.

### 3.9. Synthesis of Bis-TTFs **13a** and **13b**

A stirred mixture of 4-*p*-nitrophenyl-bis-TTFs derivatives **12a** or **12b** (4 mmol), tin (0.94 g, 8 mmol), and aqueous solution of HCl (35%) (1.8 mL, 20 mmol) in ethanol (30 mL) was refluxed for 4 h under nitrogen. During this time the initial black solution turned light yellow. The solution was then concentrated *in vacuo* and treated with an aqueous solution (100 mL) of sodium hydroxide (0.1 M) and extracted with ether. The organic phase was washed with water, dried (MgSO_4_), and concentrated *in vacuo*. The product was subjected to column chromatography on silica gel (CH_2_Cl_2_), affording the expected compounds **13a** and **13b** as powder.

p*-Aminophenyl bis-tetrathiafulvalene* ***13a***: Yield = 52%; TLC: Rf = 0.62 (CH_2_Cl_2_); dark-orange powder, mp = 138 °C; ^1^H NMR (CDCl_3_) δ ppm: 1.53 (s, 6H, C=CMe); 3.50 (t, 8H, SCH_2_, *J* = 7.38 Hz); 3.55–3.85 (m, 2H, NH_2_); 6.72 (s, 1H, C=CH); 6.60 (d, 2H_arom_, *J* = 8.47 Hz); 7.09 (d, 2H_arom_, *J* = 8.37 Hz); M.S: (NOBA, FAB > 0): 709 [M + H]^+^; M = 708; Anal. Calcd for C_24_H_23_S_12_N: C, 40.70; H, 2.98; S, 54.33; found: C, 40.97; H, 3.09; S, 54.04.

p*-Aminophenyl bis-tetrathiafulvalene* ***13b***: Yield = 48%; TLC: Rf = 0.62 (CH_2_Cl_2_); dark-orange powder, m.p. = 132 °C; ^1^H NMR (CDCl_3_) δ ppm: 1.54 (s, 6H, C=CMe); 2.44 (m, 4H, SCH_2_*CH_2_*CH_2_S); 2.95 (t, 8H, SCH_2_); 3.50–3.80 (m, 2H, NH_2_); 6.70 (s, 1H, C=CH); 6.60 (d, 2H_arom_, *J* = 8.37 Hz); 7.09 (d, 2H_arom_, *J* = 8.49 Hz); M.S: (NOBA, FAB > 0): 737 [M + H]^+^; M = 736; Anal. Calcd for C_25_H_25_S_12_N: C, 42.41; H, 3.42; S, 52.26; found: C, 42.70; H, 3.54; S, 52.10.

### 3.10. Synthesis of Bis-TTFs **14a** and **14b**

To a stirred solution of 4-aminophenyl-bis-TTF **13a** or **13b** (3 mmol) and of iodomethane (0.75 mL, 12 mmol) in acetone (15 mL) under nitrogen, K_2_CO_3_ (0.83 g, 6 mmol) was added. After 4 days of stirring at room temperature, the precipitate obtained was filtered, washed with acetone, and then extracted with CH_2_Cl_2_. The organic phase was dried (MgSO_4_) and concentrated *in vacuo*, providing the expected compounds **14a** and **14b** as dark orange powder.

p*-Dimethylaminophenyl bis-tetrathiafulvalene* ***14a***: Yield = 76%; TLC: Rf = 0.78 (CH_2_Cl_2_); orange powder, mp = 182 °C; ^1^H NMR (CDCl_3_) δ ppm: 1.53 (s, 6H, C=CMe); 3.35 (s, 6H, NMe_2_); 3.51 (t, 8H, SCH_2_, *J* = 7.37 Hz); 6.61 (s, 1H, C=CH); 7.71 (d, 2H_arom_, *J* = 8.85 Hz); 8.00 (d, 2H_arom_, *J* = 8.90 Hz); M.S: (NOBA, FAB > 0): 737 [M + H]^+^; M = 736; Anal. Calcd for C_26_H_27_S_12_N: C, 42.41; H, 3.42; S, 52.26; found: C, 42.13; H, 3.28; S, 52.57.

p*-Dimethylaminophenyl bis-tetrathiafulvalene* ***14b***: Yield = 75%; TLC: Rf = 0.78 (CH_2_Cl_2_); orange powder, mp = 176 °C; ^1^H NMR (CDCl_3_) δ ppm: 1.54 (s, 6H, C=CMe); 2.43 (m, 4H, SCH_2_*CH_2_*CH_2_S); 2.96 (t, 8H, SCH_2_); 3.35 (s, 6H, NMe_2_); 6.60 (s, 1H, C=CH); 7.71 (d, 2H_arom_, *J* = 8.85 Hz); 8.00 (d, 2H_arom_, *J* = 8.90 Hz); M.S: (NOBA, FAB > 0): 765 [M + H]^+^; M = 764; Anal. Calcd for C_27_H_29_S_12_N: C, 44.00; H, 3.82; S, 50.34; found: C, 43.76; H, 3.63; S, 50.71.

## 4. Conclusions

In summary, we have successfully prepared via Wittig-type, cross-coupling, reduction and alkylation synthetic strategies some new mono-TTFs and bis-TTFs containing nitrophenyl, aminophenyl or dimethylaminophenyl groups. All donors synthesized during the course of this work have been characterized by cyclic voltammetry and their oxidation potentials were determined by cyclic voltammetry. Charge transfer complexes of the donors with TCNQ were prepared and the electrical conductivity of these materials was measured, some CTC are conductive.

## Figures and Tables

**Table 1:** Oxidation potential of mono- and bis-tetrathiafulvalenes (bis-TTF)s.

Donor	*E*^1^_ox_ (mV)	*E*^2^_ox_ (mV)	*E*^3^_ox_ (mV)	Δ*E*_ox_ (mV)
**9**	652	988	-	336
**10a**	643	980	-	337
**10b**	640	977	-	337
**11a**	553	687	1018	465
**11b**	550	684	1014	464
**12a**	538	673	1004	466
**12b**	536	670	1001	465
**13a**	514	646	978	464
**13b**	512	643	974	462
**14a**	530	665	996	466
**14b**	528	663	994	466

**Table 2:** Electrical conductivity and melting points of charge transfer complexes.

Complex	σ_RT_ (S cm^−1^)	mp (°C)
**9-TCNQ**	1.30 × 10^−2^	265
**10a-TCNQ**	3.45 × 10^−1^	215
**10b-TCNQ**	8.60 × 10^−1^	209
**11a-TCNQ**	4.36 × 10^−3^	231
**11b-TCNQ**	6.82 × 10^−3^	226
**12a-TCNQ**	0.27 × 10^−3^	274
**12b-TCNQ**	2.85 × 10^−3^	268
**13a-TCNQ**	4.35 × 10^−4^	234
**13b-TCNQ**	7.20 × 10^−4^	228
**14a-TCNQ**	0.54 × 10^−6^	247
**14b-TCNQ**	1.30 × 10^−6^	239
